# Arthroscope-assisted reduction of humeral head impression fracture: a case report

**DOI:** 10.1093/jscr/rjac476

**Published:** 2022-11-26

**Authors:** Takuma Kachi, Hitoshi Shitara, Tsuyoshi Ichinose, Tsuyoshi Sasaki, Noritaka Hamano, Hirotaka Chikuda

**Affiliations:** Department of Orthopaedic Surgery, Gunma University Graduate School of Medicine, Maebashi, Gunma, Japan; Department of Orthopaedic Surgery, Gunma University Graduate School of Medicine, Maebashi, Gunma, Japan; Department of Orthopaedic Surgery, Gunma University Graduate School of Medicine, Maebashi, Gunma, Japan; Department of Orthopaedic Surgery, Gunma University Graduate School of Medicine, Maebashi, Gunma, Japan; Department of Orthopaedic Surgery, Gunma University Graduate School of Medicine, Maebashi, Gunma, Japan; Department of Orthopaedic Surgery, Gunma University Graduate School of Medicine, Maebashi, Gunma, Japan

## Abstract

We experienced a case of humeral head impression fracture accounting for approximately 20% of the anterior articular surface. Open reduction and internal fixation of the proximal humeral fracture combined with arthroscope-assisted reduction and internal fixation of the humeral head impression fracture were performed, and good clinical and radiographic outcomes were obtained. Untreated impression fracture may be a potential risk for subluxation or osteoarthritis. However, our arthroscopic approach is minimally invasive and allows for the prevention of these risks.

## INTRODUCTION

Proximal humeral head fracture accounts for 4–5% of all fractures [1]. When the humeral head impression involves between 20 and 40%, a modified McLaughlin procedure [2] is considered to stabilize the shoulder joint. However, the loss of range of motionof external rotation may occur with the transfer of the subscapularis [3]. If the impression area is small, non-operative treatment is selected, but osteoarthritic changes may occur in the future [4]. The present case was a young patient with a humeral head impression fracture that occupied the anterior 20% of the humeral head. We treated these fractures with the concept of preventing an invasion of the subscapularis. This case report describes the surgical technique and usefulness of arthroscopic-assisted humeral head impression fracture reduction. We referred to previous reports [5, 6] describing arthroscopic reduction techniques.

## CASE PRESENTATION

**Figure 1:**
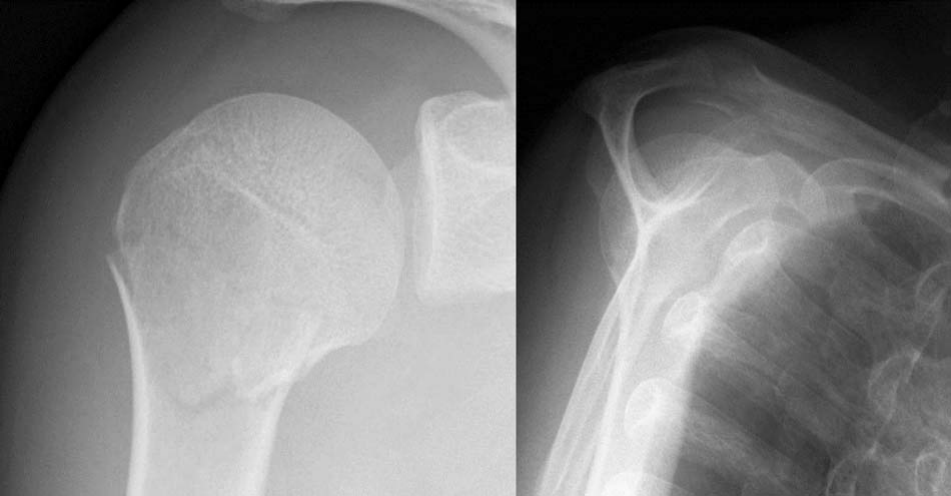
Original X-ray showing surgical neck fracture.

**Figure 2:**
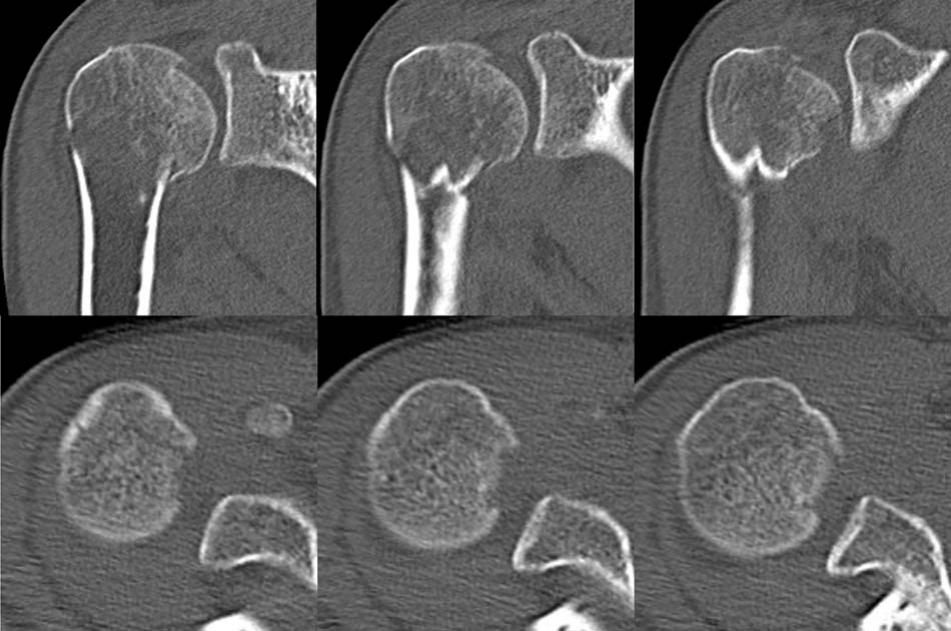
Original CT scan showing impression fracture and surgical neck fracture.

**Figure 3:**
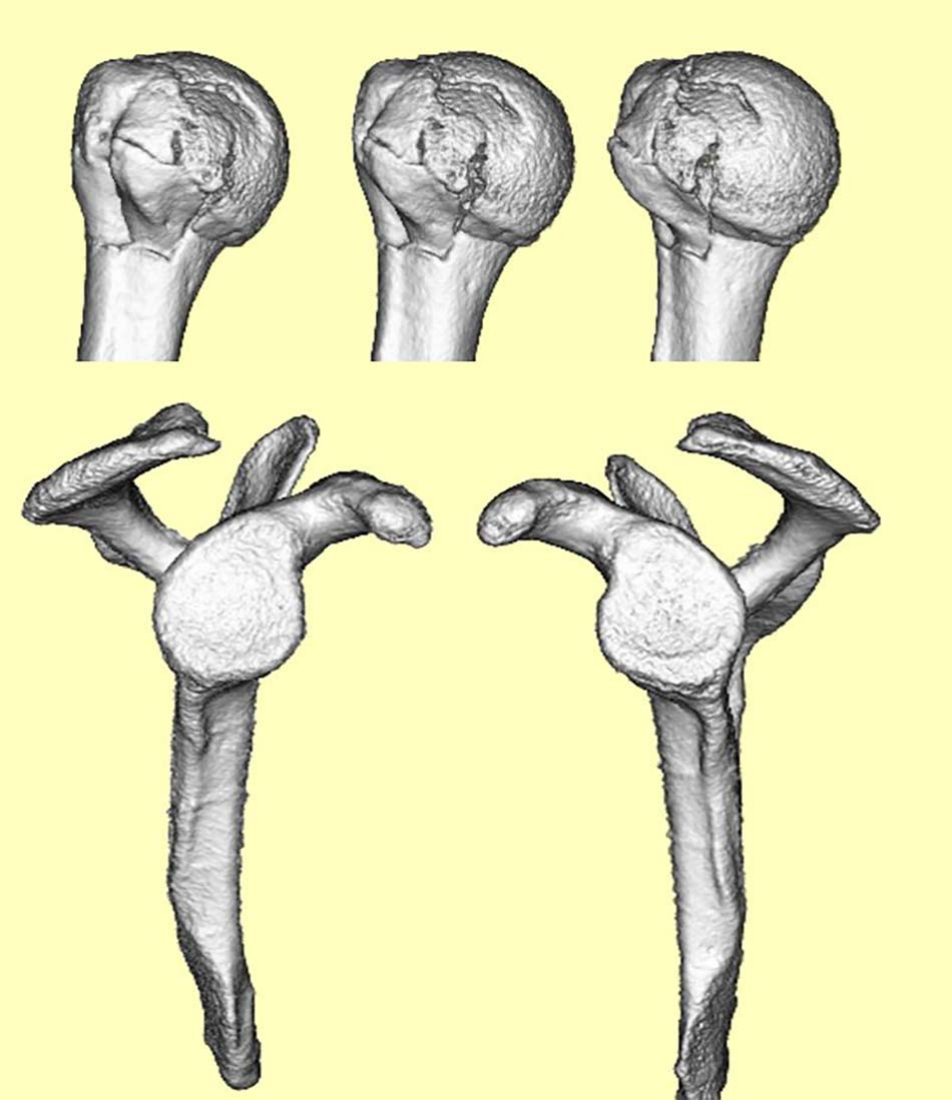
3D-CT scan showing approximately 20% of articular surface of the right proximal and no glenoid defect, indicating no posterior shoulder dislocation.

A 39-year-old man was transported via ambulance after being hit by a car. The radiographs revealed a one-part proximal humeral fracture with displacement at the surgical neck (Fig. 1). However, computed tomography (CT) revealed humeral head impression fracture and lesser tuberosity fracture in addition to a surgical neck fracture (Fig. 2). In addition, 3D-CT revealed that the collapse occupied approximately 20% of the articular surface with no glenoid defect (Fig. 3). Therefore, we first performed arthroscopic-assisted reduction and internal fixation of the humeral head impression fracture, followed by open reduction and internal fixation (ORIF) of the proximal humeral fracture.

**Figure 4:**
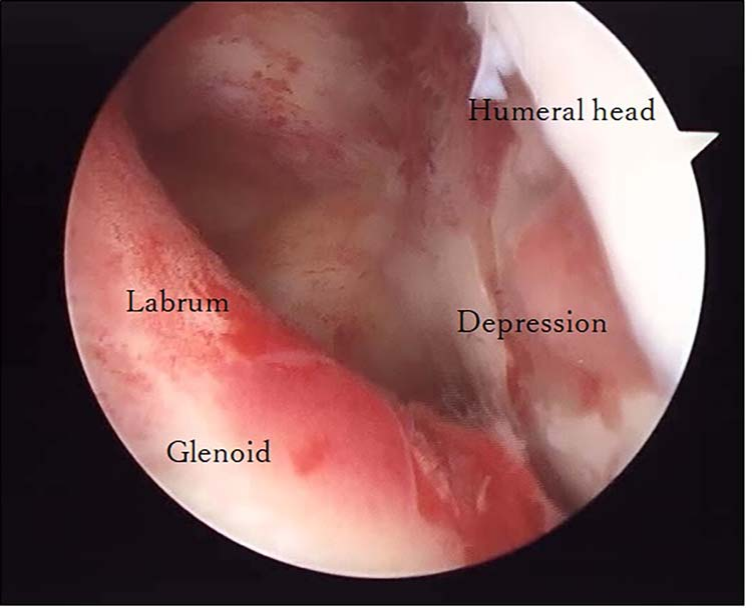
Posterior arthroscopy of the right shoulder joint. The right is the humeral head side, and the left is the glenoid side. Impression of the humeral head was noted, but the posterior labrum was not damaged.

**Figure 5:**
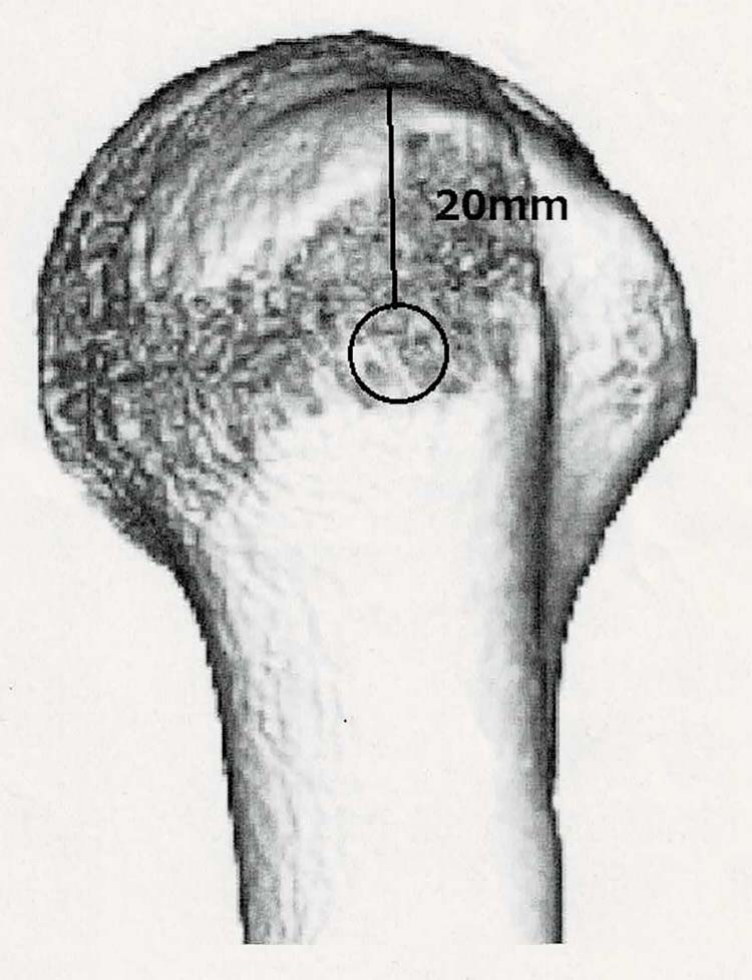
To correctly place the cortical window, we referenced Marco’s report. We took a distance of 20 mm from merkmal against they took 25 mm.

**Figure 6:**
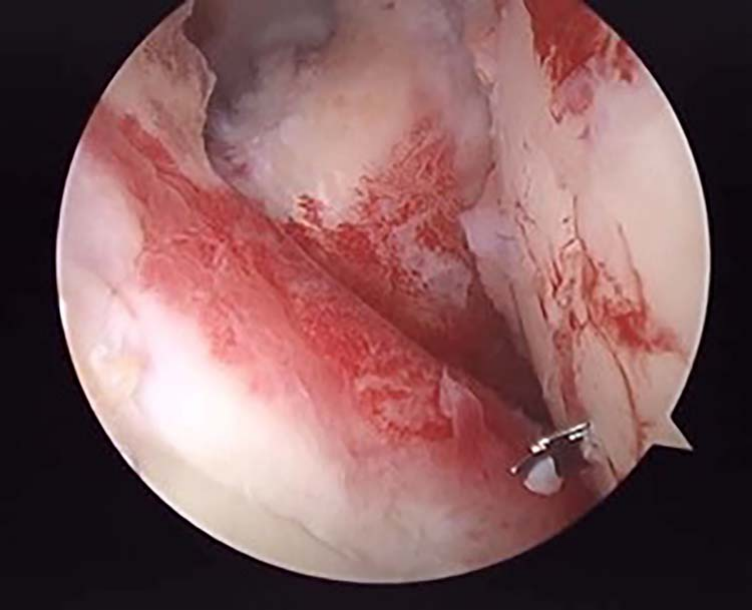
Posterior arthroscopy of the right shoulder joint showing a reduced depressed surface and the Kirschner wire for reduction guide.

The operation was performed under general anesthesia with an ultrasound-guided brachial plexus block in the beach chair position. First, we performed intra-articular observation by arthroscopy with a 30° scope. Through a posterior portal, we confirmed incongruence of the articular surface of the humeral head, similar to the preoperative findings in 3D-CT (Fig. 4). On the other hand, the posterior joint labrum and glenoid rim were intact. After evaluating the intra-articular lesion, we performed arthroscopic reduction for the impression fracture at the humeral head via the arthroscopic method [5,6]. We split the deltoid muscle and exposed the lateral surface of the humerus. We inserted a Kirschner wire as a reduction guide 2 cm distal from the upper border of the greater tuberosity and 2 cm behind the bicipital groove (Fig. 5). Kirschner wire was inserted to exit the impression lesion while checking the articular surface with an arthroscope (Fig. 6). We created a cortical window (approximately 10 × 10 mm) using a cannulated drill with the Kirschner wire as a guide. To reduce the impression, we pushed the back of the impression using the flat side of the canulated cancellous screw (CCS) drill. After the reduction, artificial bone was inserted from the cortical window to the subchondral bone region to provide support for the depressed area. We then performed a procedure to insert a support screw into the articular surface. The deltopectoral approach was used. First, we dissected the subacromial bursa entirely while visualizing the conjoint tendon. Next, we detached the long head of the biceps at the bicipital groove and sutured it to the pectoralis major muscle. Two CCSs were inserted from the bicipital groove to under the repaired humeral head as support for the reduction site. Finally, after arthroscopic reduction, we performed ORIF for surgical neck fracture using a PHILOS Plate™ (DePuy Synthes).

**Figure 7:**
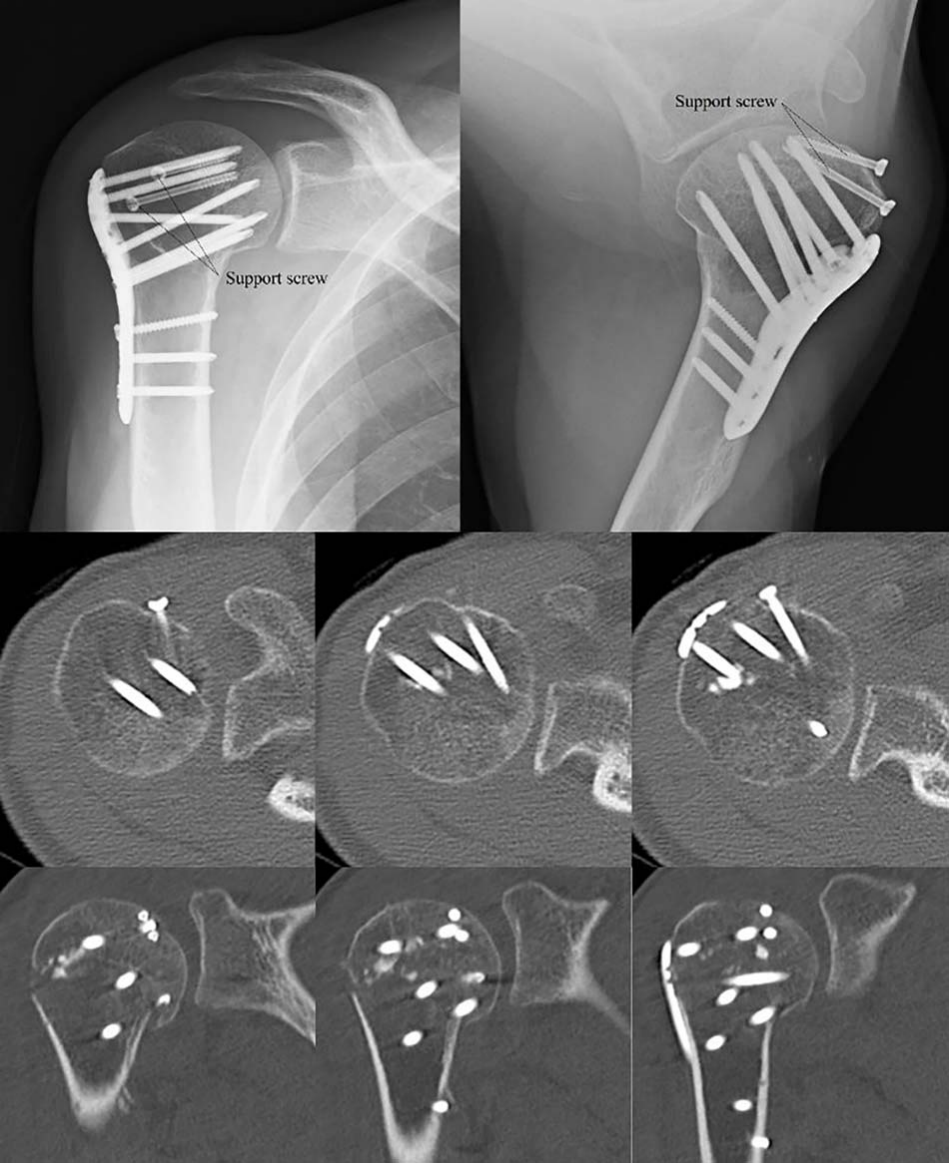
Postoperative X-ray and CT scan showing sustained anatomical reconstruction of articular surfaces and no signs of osteoarthritis.

**Figure 8:**
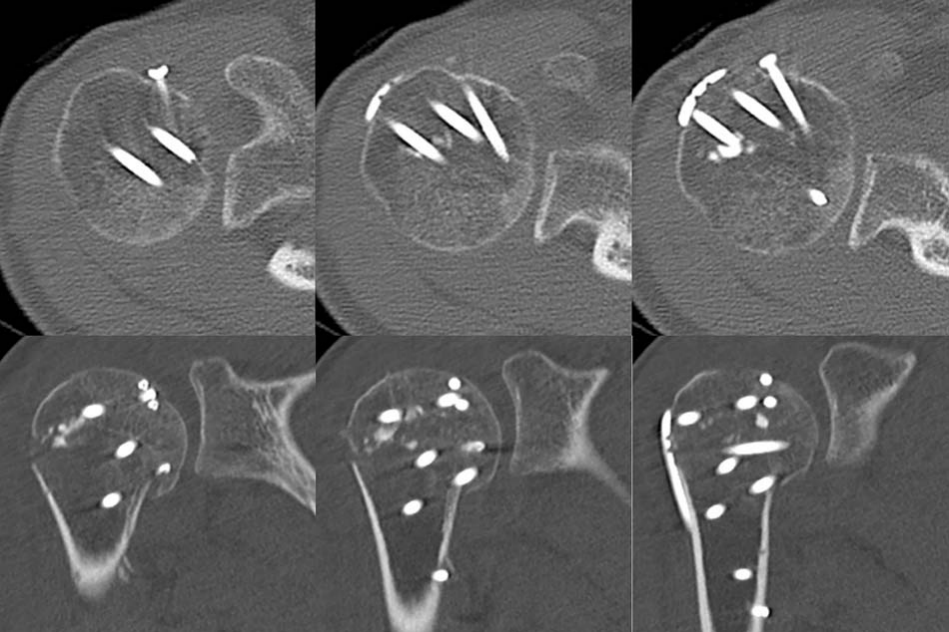
Two years later, pre-implant removal CT showed that the reduction in the depressed surface had been maintained with no obvious arthritic changes.

**Figure 9:**
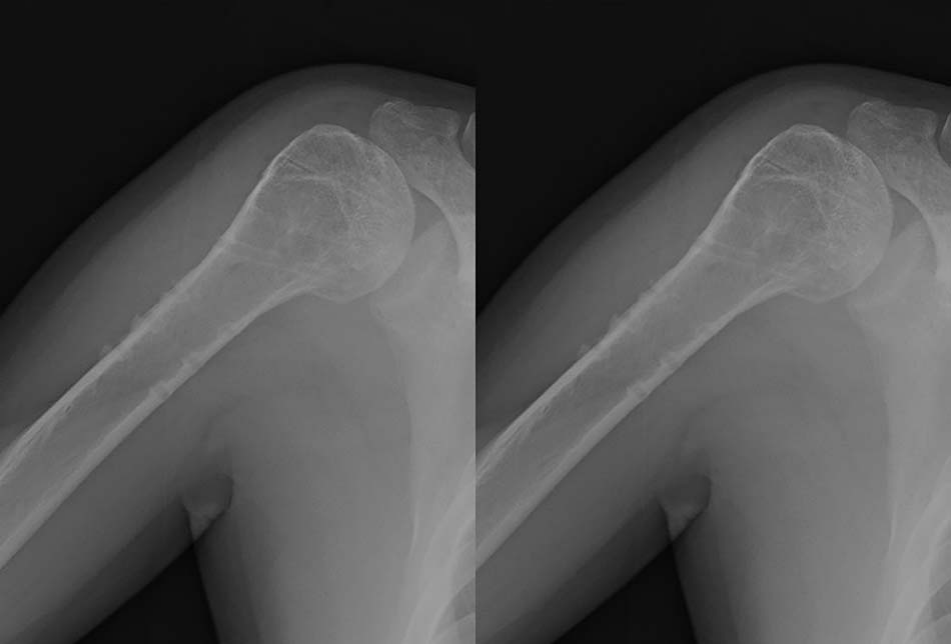
Post-implant removal X-ray showing that the alignment had been maintained.

**Figure 10:**
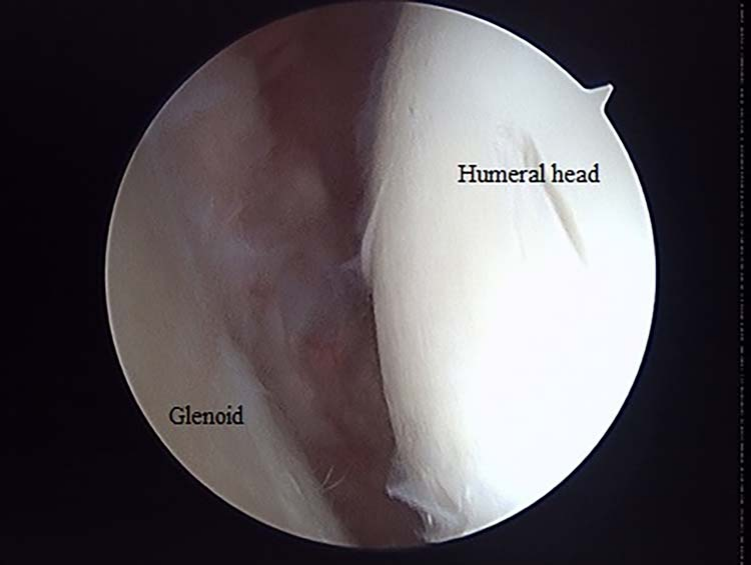
Posterior endoscopy of the shoulder joint showing no reimpression or exposure of subchondral bone.

Postoperative radiographic and CT evaluations showed reduced fractures on the humeral head and surgical neck (Fig. 7). At 24 months of postoperative follow-up, radiographic and CT evaluations showed no signs of osteoarthritis or reimpression (Fig. 8). Two years after the primary surgery, we performed implant removal at the patient’s request (Fig. 9). When performing implant removal, we additionally performed a second-look evaluation with arthroscopy to assess the degree of healing in the joint. We noted no impression or exposure of cartilage callus on either side of the humeral head or glenoid (Fig. 10).

## DISCUSSION

In humeral head impression fractures, there are no clear criteria regarding the risk of developing osteoarthritis due to the impression area; however, if the deformity of the humeral head is left unrepaired, there may be a potential risk of osteoarthritic changes [4]. On the other hand, the problem of joint instability depends on the extent of the area [7]. If the area is greater than 20%, there is a risk of dislocation when the major articular fragment is unstable.

When the impression involves 20–40%, a modified McLaughlin procedure [2] is considered to stabilize the shoulder joint. In this case, the impression size was 20%; thus, we selected surgical reduction, based on the findings of a previous study [8], and further taking into account the patient’s age and activity level. However, there is concern about the limitation of the external rotation angle due to invasion of the subscapularis [3]. Furthermore, the patient had a concomitant proximal humerus fracture, so an inferior outcome may be expected [2] with the modified McLaughlin procedure.

Our purpose was to repair the impression with minimal invasion of the joint and rotator cuff. An arthroscope-assisted reduction [5, 6], as performed in this case, is minimally invasive and not difficult, and allows for the prevention of osteoarthritis and dislocation without transfer of the subscapularis or joint replacement.

The main limitation of this case, in common with the previous reports [3, 7], is that arthroscopic repair is not indicated when the impression area is >50%, in such cases, prosthesis should be considered.

The decision on how to treat a depressed humeral head fracture must be jointly made by the orthopedic surgeon and the patient. In such cases, it is of great benefit to patients that we—as orthopedic surgeons—can provide a minimally invasive arthroscopic procedure.

The authors, their immediate family and any research foundation with which they are affiliated did not receive any financial payments or other benefits from any commercial entity related to the subject of this article.
